# 基于共价有机框架材料TpPa-NH_2_-Glu的高效液相色谱固定相用于分离手性化合物

**DOI:** 10.3724/SP.J.1123.2022.06012

**Published:** 2023-02-08

**Authors:** Hualin LIU, Yanan LI, Min ZI, Zheng CHEN, Aihong DUAN, Liming YUAN

**Affiliations:** 云南师范大学化学化工学院，云南 昆明 650500; School of Chemistry and Chemical Engineering，Yunnan Normal University，Kunming 650500，China

**Keywords:** 共价有机框架材料, 手性固定相, 手性分离, 高效液相色谱, covalent organic framework materials （COFs）, chiral stationary phase （CSP）, chiral separation, high performance liquid chromatography （HPLC）

## Abstract

共价有机框架材料（COFs）是一类由多齿有机单元通过共价键连接而成的新兴孔晶体材料。通过合成后修饰所得到的COFs通常具有较高的结晶度与孔隙率，并且在手性拆分、不对称催化与色谱分析等领域具有良好的应用价值。该文用1，3，5-三甲醛间苯三酚与2-硝基-1，4-苯二胺合成TpPa-NO_2_，对其还原得到TpPa-NH_2_，然后通过合成后修饰策略将D-葡萄糖修饰到该材料上，得到手性材料TpPa-NH_2_的D-葡萄糖衍生物（TpPa-NH_2_-Glu）。利用“网包法”将其固载在球形硅胶表面用作高效液相色谱固定相并制备了液相色谱柱，分别以正己烷-异丙醇（9∶1， v/v）或甲醇-水（9∶1， v/v）为流动相，流速0.5 mL/min，成功拆分了16种包括多个手性药物的外消旋体和2种苯系位置异构体*o*，*m*，*p*-硝基苯胺和*o*，*m*，*p*-碘苯胺。在甲醇-水（9∶1， v/v）流动相条件下，拆分了5种外消旋体，其中盐酸普萘洛尔、华法林、美托洛尔达到了基线分离；在正己烷-异丙醇（9∶1， v/v）流动相条件下，拆分了11种外消旋体，其中2-溴丙酸乙酯、3-丁炔-2-醇达到了基线分离。此外还探究了温度对TpPa-NH_2_-Glu液相色谱柱的影响以及TpPa-NH_2_-Glu液相色谱柱的重复性，结果表明，温度对TpPa-NH_2_-Glu所制备的高效液相色谱柱影响不大，且TpPa-NH_2_-Glu所制备的高效液相色谱柱具有良好的重复性，其相对标准偏差（RSD）分别为1.55%和1.46%。实验结果表明，TpPa-NH_2_-Glu对手性化合物有良好的拆分能力。

## Abstract (en)

Covalent organic frameworks （COFs） are an emerging class of porous crystalline materials composed of multidentate organic units connected by covalent bonds. COFs have been demonstrated to exhibit great potential and research value in many fields， including gas storage and separation， photoelectric devices， fluorescence sensors， catalysis， drug delivery， dye and pollutant adsorption， and electronic devices， and so on. The COFs obtained by post-synthesis modification tend to exhibit high crystallinities and porosities， thereby rendering them suitable materials for use in the fields of chiral resolution， asymmetric catalysis， and chromatography. In this work， TpPa-NO_2_ was synthesized from 1，3，5-tricarbaldehyde phloroglucinol and 2-nitro-1，4-phenylenediamine， which was then reduced to TpPa-NH_2_. Subsequently， this material was modified with D-glucose via a post-synthesis modification strategy to obtain the TpPa-NH_2_-Glu. TpPa-NH_2_-Glu were characterized by nuclear magnetic resonance （NMR） spectroscopy， Fourier transform-infrared （FT-IR） spectroscopy， X-ray powder diffraction （XRD） analysis， etc. In the XRD pattern， the peaks observed at 4.7°， 8.1°， 11.1°， and 27° were attributed to the TpPa-NH_2_-Glu， and these peaks are consistent with previous reports， thereby confirming the successful synthesis of this derivative. In addition， circular dichroism experiments indicated that the TpPa-NH_2_-Glu exhibited a Cotton effect， further confirming the chiral COF was prepared. Subsequently， this material was immobilized on the surface of spherical silica gel particles via the net-wrapping method to prepare a stationary phase for high performance liquid chromatographic column. Using *n*-hexane-isopropanol （9∶1， v/v） or methanol-water （9∶1， v/v） as mobile phases at a flow rate of 0.5 mL/min， 16 racemates and two benzene-based positional isomers （*o*，*m*，*p*-nitroaniline and *o*，*m*，*p*-Iodoaniline） were successfully resolved by this chiral column. In addition， under methanol-water （9∶1， v/v） mobile phase conditions， five racemates were separated， among which propranolol hydrochloride， warfarin， and metoprolol reached baseline separation. Furthermore， under *n*-hexane-isopropanol （9∶1， v/v） mobile phase conditions， 11 racemates were resolved， among which ethyl 2-bromopropionate and 3-butyn-2-ol reached baseline separation. Meanwhile， the effect of temperature on the TpPa-NH_2_-Glu liquid chromatography column and the repeatability of the TpPa-NH_2_-Glu liquid chromatography column were also explored. The HPLC column prepared by TpPa-NH_2_-Glu had good repeatability， and its relative standard deviation （RSD） was 1.55% and 1.46%， respectively. It is demonstrated that the TpPa-NH_2_-Glu material has good resolution ability for chiral compounds.

在自然界的生命活动中，都有手性识别这个重要因素存在，从物种和数目上来研究，有机生命体几乎都是由手性分子组成的^[1]^。特别是具有手性的药物，虽然同一化合物的两种光学异构体化学特性以及物理特性几乎相同，但因其药物对生命作用的靶位或受体是核酸和蛋白质等具有手性的生物大分子，因此对与其相作用的药物分子也具有手性选择性，这也导致手性药物在生命体的生命代谢中通常呈现较大的药理、毒理差异性，而在药物动力学、药效学方面也具有不同的功能作用^[2,3]^。目前用于临床的药物有4000余种，其中约一半是人工合成药物，而这些合成药物中又有近50%是外消旋体^[4]^。随着人们对单一手性药物需求的增长，探索获得纯对映体的有效途径变得越来越迫切，因此，探索有效分离对映体的新材料或新方法实现对映体的有效分离具有重要意义。

共价有机框架材料（COFs）是由有机小分子单体通过共价键连接形成的一种具有二维或三维结构的新型多孔晶体材料^[5⇓⇓-8]^。COFs材料作为一种纯有机的晶态多孔材料，具有很多优点，比如低密度、多变的结构和化学性质、良好的结晶度、稳定性以及可以调节的功能^[9]^。因此，COFs材料是研究手性的一个很好的平台，在手性催化、手性分离、样品预处理^[10]^等领域具有潜在的应用。目前已经有一系列功能化COFs见于报道，如TpPa系列的COFs材料，它们通过溶剂热法^[11]^，利用醛胺席夫碱缩合反应合成，通过后合成修饰法（PSM）进行改性，修饰上手性基团。2016年，Yan等^[12]^将手性羧酸衍生物引入到COFs结构中。将手性二乙酰-L-酒石酸酐与1，3，5-三甲酰基间苯三酚发生酯化反应，酒石酸酐开环后得到含3个甲酰基的三羧酸衍生物CTp。以CTp为C3对称单体，分别与1，4-苯二胺（Pa-1）、2，5-二甲基对苯二胺（Pa-2）和联苯胺（BD）的C2对称单体发生缩合反应，自下而上地制备了CTpPa-1、CTpPa-2和CTpBD这3种羧酸类手性COFs材料。所制备的3种羧酸类手性COFs材料具有良好的手性分离性能，上海交通大学崔勇团队^[13]^于2018年合成了第一例具有手性醇特征的三维手性COFs材料。仍然采用C2对称的四芳基-1，3-二氧戊环-4，5-二甲醇为手性片段，衍生化得到（*R*，*R*）-四苯甲醛为醛基单元，与四面体构型的四（4-苯基）甲烷为氨基单元，发生缩合反应制备了（*R*，*R*）-COFs，进一步氧化得到酰胺官能团链接的（*R*，*R*）-COFs。2019年，Yuan等^[14]^首次将手性*β*-环糊精（*β*-CD）修饰到COFs材料中，得到了基于COFs的混合基质膜（MMMs），用于选择性传输氨基酸。2022年4月，Ma等^[15]^报告了一种新的羟基官能化共价有机骨架TzDHNDA与[（1-苯乙基）氨基]乙酸（PEAA）通过酯化反应构建PEAA官能化COF材料TzDHNDA-PEAA。

本文通过合成TpPa-NO_2_，对其还原得到TpPa-NH_2_，使用PSM将D-葡萄糖修饰到该材料上，得到手性材料TpPa-NH_2_的D-葡萄糖衍生物（TpPa-NH_2_-Glu）。采用X射线粉末衍射分析（PXRD）、红外光谱分析（FT-IR）、扫描电镜（SEM）、氮气吸附测试、圆二色谱（CD）对合成的手性COFs材料进行了表征，然后将合成的TpPa-NH_2_-Glu与球形硅胶混合均匀后作为高效液相色谱固定相，并采用高压匀浆法成功制备了高效液相色谱柱。在一定条件下，用TpPa-NH_2_-Glu液相色谱柱对外消旋化合物和常见的苯系位置异构体进行拆分，最终有16种外消旋体和2种苯系位置异构体有不同的拆分效果，表明手性TpPa-NH_2_-Glu用作液相色谱固定相具有很大的应用前景。

## 1 实验部分

### 1.1 仪器、试剂与材料

Elite P230 Ⅱ高效液相色谱仪配有AT-330柱温箱（大连依利特公司）； S-3000N扫描电子显微镜（日本Hitachi公司）； Chirasca圆二色谱仪（英国Applied Photophysics公司）； SX2-4-10马弗炉（上海意丰电炉有限公司）； DJ-1大功率磁力搅拌（常州申光仪器有限公司）； TDZ5-WS台式低速离心机（湖南湘仪实验室仪器开发有限公司）； 1666型液相色谱装柱机（美国Alltech有限公司）； DHG-9035A鼓风电热干燥箱（上海恒以科学仪器有限公司）； ASAP2020 M+C氮气吸附仪（美国Micromeritics公司）； DZF型真空干燥箱（北京科伟永兴仪器有限公司）。

间苯三酚（纯度98%）购于郑州阿尔法化工有限公司；三氟乙酸（纯度97%）、邻硝基苯胺（纯度99%）、碘化钾（纯度98%）、高碘酸钾（纯度99%）、六亚甲基四胺（纯度97%）、位置异构体*o*，*m*，*p*-碘苯胺和*o*，*m*，*p*-硝基苯胺（纯度99%）均购于上海Aladdin生化科技股份有限公司；氯化钠（纯度≥99%）、D-葡萄糖（纯度99%）均购于天津市风船化学试剂科技有限公司；氘代氯仿（CDCl_3_）、单体2-硝基-1，4-苯二胺（Pa）（纯度≥98%）、三乙胺（纯度≥99%）、二茂铁二氯化钯（纯度99%）、均三甲苯（纯度99%）、无水SnCl_2_（纯度99%）均购于上海Aadmas试剂有限公司；1，4-二氧六环（纯度98%）以及外消旋化合物盐酸普萘洛尔、华法林、美托洛尔、佐匹克隆、精氨酸、阿替洛尔、吲哚洛尔、3-苄氧基-1，2-丙二醇、3-丁炔-2-醇、2-丁胺、1，3-丁二醇、2-溴丙酸乙酯、丙二醇单甲醚、1，2-环氧辛烷、环氧溴丙烷、D，L-2-苯基丙醛（纯度99%）均购于美国Sigma-Aldrich公司；硅胶（UniSil 5-100）购于苏州纳微科技有限公司。

### 1.2 1，3，5-三甲醛间苯三酚（Tp）的合成

根据已有文献^[16]^合成了1，3，5-三甲醛间苯三酚，分别取7.549 g六亚甲基四胺（54 mmol）和3.007 g间苯三酚（24.5 mmol）加入到三颈烧瓶中，真空脱气后，在氮气环境下，加入90 mL三氟乙酸，在100 ℃条件下搅拌3 h后，再取150 mL 0.3 mol/L的盐酸溶液加入到反应容器中，再反应2.5 h。冷却至室温，用二氯甲烷萃取后，取下层有机相，蒸发溶剂后，用甲醇抽滤洗涤产物，得到灰白色固体1，3，5-三甲醛间苯三酚。

### 1.3 TpPa-NH_2_的合成

根据已有文献^[17]^合成了TpPa-NH_2_，分别称取63 mg上步合成的Tp （0.3 mmol）、48 mg 2-硝基-1，4-苯二胺（Pa-NO_2_， 0.45 mmol）、1，3，5-均三苯甲醛（0.75 mL）、1，4-二氧六环（0.75 mL）和0.5 mL 3 mol/L醋酸于Pyrex耐热玻璃管中，超声至其混合均匀，用液氮冷冻后，抽真空脱气，用酒精喷灯封管，将Pyrex耐热玻璃管在120 ℃下反应72 h，反应完成后，取出产物，依次用50 mL的丙酮、二氯甲烷洗涤数次，得到红棕色产物。取3 g SnCl_2_·2H_2_O和150 mg上述合成的TpPa-NO_2_于50 mL的二颈烧瓶中，加入5 mL无水四氢呋喃使其混合均匀，在50 ℃条件下加热回流3 h。反应完成后，将沉淀物用70 mL的1 mol/L盐酸洗涤10次，用70 mL的水洗涤3次，再用100 mL丙酮洗涤一次。取上步得到的红棕色粉末，置于合适的反应釜中，加入5 mL苯甲醚，在120 ℃下反应24 h。最后，将粉末过滤并用100 mL丙酮洗涤。得到红棕色产物TpPa-NH_2_。

### 1.4 TpPa-NH_2_-Glu的合成

称取150 mg TpPa-NH_2_、1.0 g干燥过的D-葡萄糖和20 mL的无水甲醇于二颈烧瓶中，在70 ℃条件下搅拌4 d，反应完成后，用纯水洗去未反应完的葡萄糖，烘干，得到红棕色产物。合成线路图见图1。

**图1:**
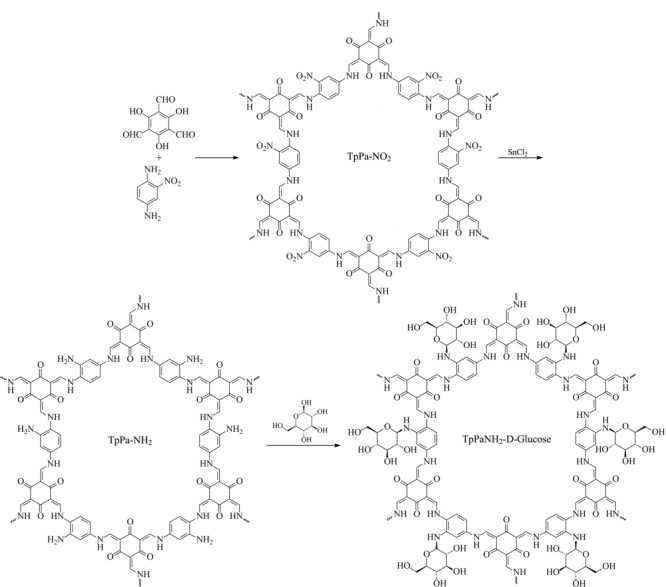
TpPa-NH_2_-Glu合成路线图

### 1.5 TpPa-NH_2_-Glu固定相的制备

#### 1.5.1 活化硅胶

称取2 g均苯三甲酰氯于100 mL容量瓶中，用正己烷定容，然后称取3.0 g球型硅胶于烧杯内，加入已配制好的溶液，浸透12 h后，将多余的溶液用滴管吸出，室温下晾干。

#### 1.5.2 网包法制备固定相

将TpPa-NH_2_-Glu用玛瑙研钵研磨，称取1.0 g哌嗪于50 mL容量瓶内，加入异丙醇和2 mL三乙胺配制成50 mL溶液，称取100 mg已研磨的TpPa-NH_2_-Glu加入到上述溶液中，超声后浸泡3~4 h，吸出多余液体，在110 ℃烘箱里加热处理15 min，备用。

#### 1.5.3 TpPa-NH_2_-Glu液相色谱柱的填充

取4.2 g制备好的TpPa-NH_2_-Glu固定相装入50 mL小烧杯中，加入36 mL正己烷-异丙醇（9∶1， v/v）溶液，搅拌均匀后快速将悬浊液倒入匀浆罐中，用正己烷-异丙醇（9∶1， v/v）作为顶替液，在35 MPa的压力下放置5 min，然后将压力降至25 MPa，再放置30 min。最后，将制备好的TpPa-NH_2_-Glu液相色谱柱接入高效液相色谱仪，用正己烷-异丙醇（9∶1， v/v）溶液冲洗2 h，待基线平稳后即可测样。

### 1.6 色谱条件

盐酸普萘洛尔、华法林、美托洛尔、佐匹克隆、精氨酸：液相色谱空柱（250 mm×4.6 mm， 5 μm， Alltech，美国）；流动相为甲醇-水（9∶1， v/v）；流速为0.5 mL/min，紫外检测波长为254 nm、210 nm，柱温为25 ℃。

其他11种外消旋化合物、*o*，*m*，*p*-碘苯胺和*o*，*m*，*p*-硝基苯胺：流动相为正己烷-异丙醇（9∶1， v/v），其他色谱条件同上。

## 2 结果与讨论

### 2.1 1，3，5-三甲醛间苯三酚的结构表征

对1，3，5-三甲醛间苯三酚进行核磁共振氢谱表征。^1^H NMR （500 MHz， CDCl_3_）： 10.18（s， 3H， CHO）， 14.15（s， 3H， OH）。MS （ESI， 50 eV） *m/z* 210 （M^+^），上述所得数据与文献报道^[16]^一致，表明已经成功合成1，3，5-三甲醛间苯三酚。

### 2.2 TpPa-NH_2_-Glu的结构表征

#### 2.2.1 X射线粉末衍射分析

采用X射线粉末衍射对按上述方法合成的TpPa-NO_2_ （图2a）、TpPa-NH_2_（图2b）和TpPa-NH_2_-Glu（图2c）进行了表征。根据文献报道，TpPa-NO_2_在低角度4.7°显示出第一个峰，对应于（100）反射面，以及8.1°、11.1°和27°处的峰，分别归因于（200）、（210）和（001）反射面，属于AA堆叠模式。由图2看出，在4.7°、8.1°、11.1°和27°均出现了衍射峰，与文献报道^[17]^一致，表明TpPa-NO_2_成功合成。经还原的TpPa-NO_2_和用D-葡萄糖修饰后的TpPa-NH_2_得到TpPa-NH_2_-Glu，它们的主要衍射峰也在4.7°、8.1°、11.1°和27°处，三者的峰形和出峰的位置基本相似，这也说明还原以及修饰后的TpPa-NO_2_的晶型结构基本保持不变。

**图2:**
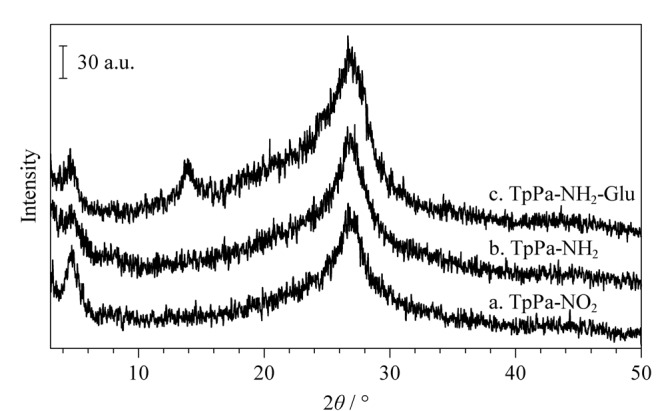
3种材料的X射线粉末衍射图

#### 2.2.2 红外光谱分析

采用红外光谱对合成的TpPa-NO_2_（图3a）、TpPa-NH_2_（图3b）和TpPa-NH_2_-Glu（图3c）进行了表征。在图3a中，1523 cm^-1^和1445 cm^-1^的吸收峰由-NO_2_振动产生。在图3b中，1523 cm^-1^和1445 cm^-1^处的峰强度减弱，说明TpPa-NO_2_中的-NO_2_被还原了。在图3c中，在1069 cm^-1^处出现C-O的特征吸收峰，与图3b对比，峰的强度增强，出现了明显的尖锐峰，说明D-葡萄糖已成功衍生在TpPa-NH_2_上。

**图3:**
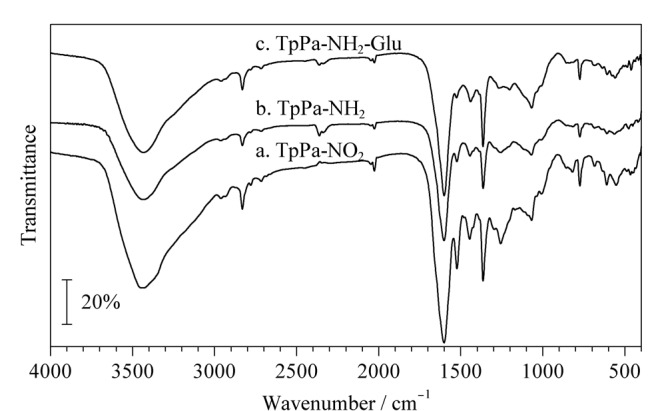
3种材料的红外光谱图

#### 2.2.3 圆二色谱分析

如图4所示，经过D-葡萄糖修饰后的TpPa-NH_2_显示出了Cotton效应，表明葡萄糖成功修饰在COFs上，使衍生后的TpPa-NH_2_具有了手性。

**图4:**
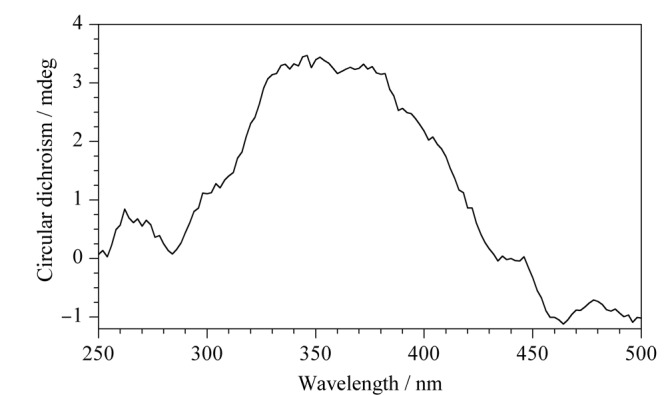
TpPa-NH_2_-Glu的圆二色谱分析

#### 2.2.4 氮气吸附测试

为测试TpPa-NH_2_-Glu比表面积、空腔体积及孔径大小，进行氮气吸附试验，结果见图5，得到TpPa-NH_2_-Glu的比表面积为218 m^2^/g，孔体积为0.94 cm^3^/g，平均孔径为1.79 nm，说明TpPa-NH_2_-Glu比表面积较大，适用于一般有机化合物的分离。

**图5:**
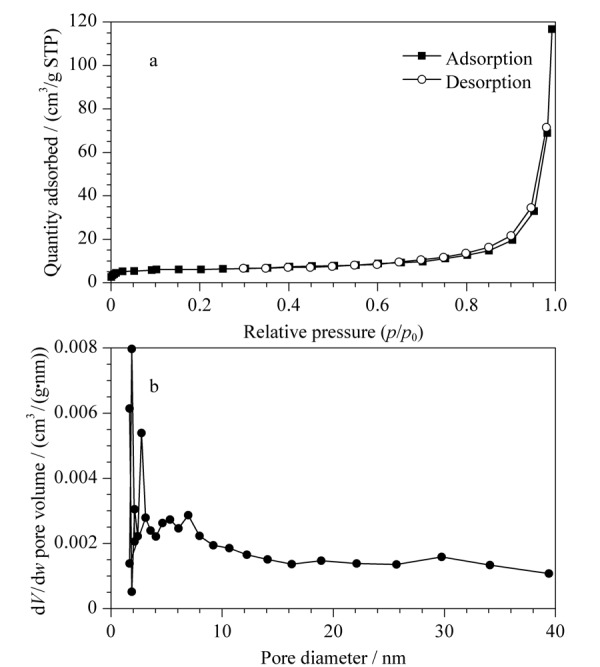
TpPa-NH_2_-Glu的（a）氮气吸附脱附等温线和（b）孔径分布曲线

#### 2.2.5 扫描电镜分析

为观察TpPa-NH_2_-Glu是否固载到硅胶上，对网包后的固定相进行扫描电镜分析。对比未网包的硅胶与网包后的固定相的扫描电镜图（见图6），可以明显看到硅胶外部网包上一层TpPa-NH_2_-Glu材料，说明通过网包法，所合成的手性COF材料成功固载在硅胶表面。

**图6:**
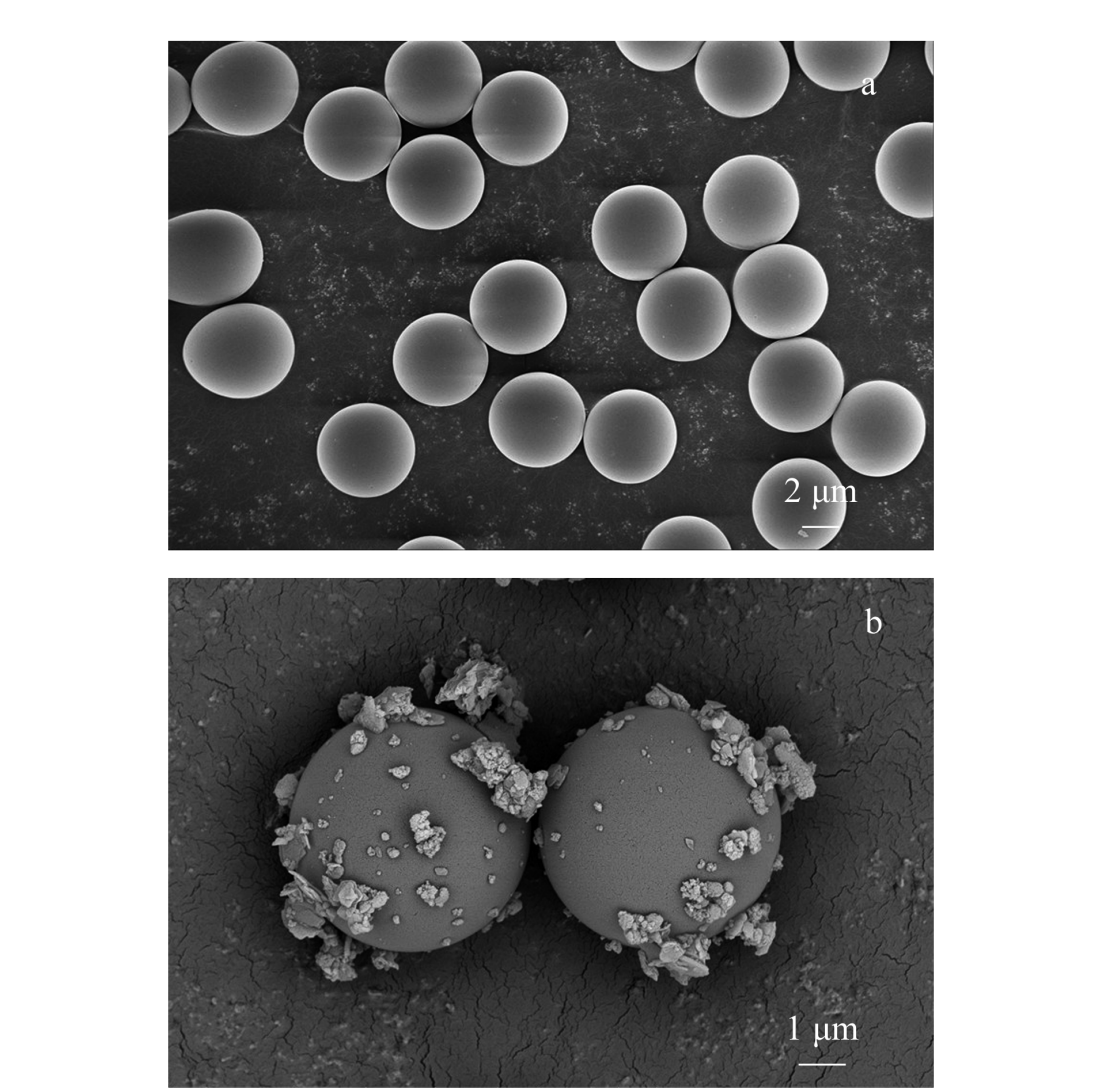
固定相扫描电镜图

### 2.3 TpPa-NH_2_-Glu液相色谱柱对外消旋体的拆分

在流动相为正己烷-异丙醇（9∶1， v/v）或甲醇-水（9∶1， v/v）、柱温为25 ℃、流速为0.5 mL/min的色谱条件下，用一系列手性化合物来考察TpPa-NH_2_-Glu液相色谱柱对外消旋体的手性拆分能力。实验结果表明，TpPa-NH_2_-Glu液相色谱柱对手性化合物具有良好的拆分效果，16种手性化合物在该手性液相色谱柱上得到了拆分。表1总结了这16种外消旋化合物的分离因子（*α*）和分离度（*R*_s_）等色谱参数，其拆分谱图见图7。

**表1:** TpPa-NH_2_-Glu液相色谱柱对16种外消旋体的拆分结果

Mobile phase	Racemate	*k*_1_	*k*_2_	*α*	*R*_s_
Methanol-	warfarin	0.52	1.20	2.29	2.60
water（9∶1，	propranolol hydrochloride	0.35	1.22	3.49	5.02
v/v）	metoprolol	0.61	2.14	3.50	3.01
	zopiclone	0.81	1.39	1.71	0.74
	arginine	0.38	0.43	1.12	0.56
*n*-Hexane-	atenolol	0.95	1.18	1.25	1.05
isopropanol	pindolol	0.89	1.16	1.31	1.10
（9∶1，v/v）	3-benzyloxy-1，2-	0.60	1.01	1.68	0.76
	propanediol				
	3-butyn-2-ol	0.50	0.93	1.86	1.59
	2-butylamine	0.64	0.94	1.47	1.02
	1，3-butanediol	0.69	1.00	1.45	1.08
	ethyl 2-bromopropionate	0.88	1.43	1.64	2.49
	propylene glycol	0.99	1.17	1.18	0.85
	monomethyl ether				
	1，2-epoxyoctane	0.94	1.06	1.13	0.40
	epoxy bromopropane	1.10	1.65	1.50	1.25
	D，L-2-phenylpropanal	1.47	1.68	1.14	0.50

*k*：retention factor；*α*：separation factor；*R*_s_：resolution.

**图7:**
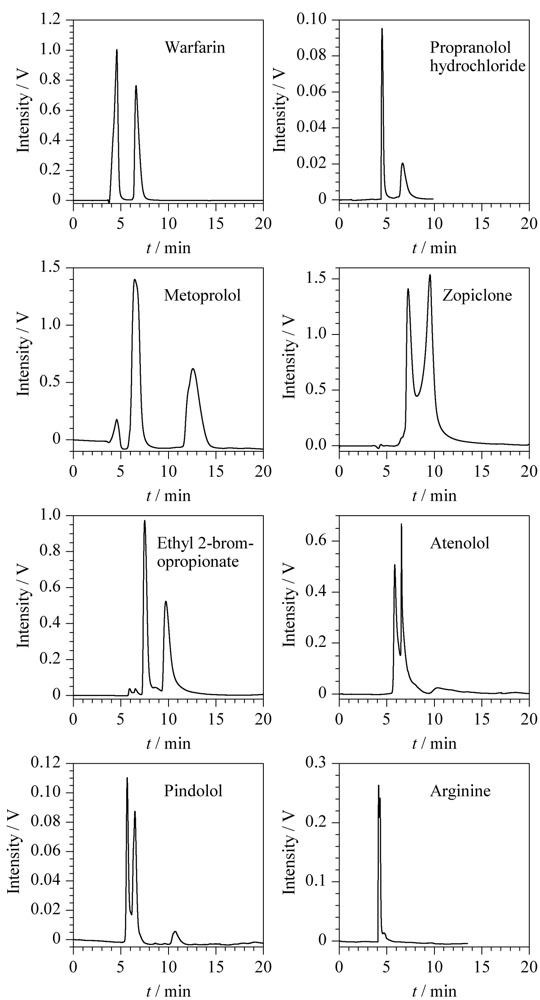
手性化合物在TpPa-NH_2_-Glu液相色谱柱上的拆分色谱图

### 2.4 TpPa-NH_2_-Glu液相色谱柱对苯系位置异构体的拆分

在流动相为正己烷-异丙醇（9∶1， v/v）、柱温为25 ℃、流速为0.5 mL/min的色谱条件下，TpPa-NH_2_-Glu液相色谱柱对位置异构体*o*，*m*，*p*-碘苯胺、*o*，*m*，*p*-硝基苯胺也进行了分离研究（见图8）。因位置异构体的分子结构、大小各不相同，与固定相接触时产生的效果存在差异，在苯系位置异构体中，其邻、间、对位的长宽比不一样，与固定相的作用力大小也不一样，其保留时间不同，从而使位置异构体分离。表2总结了*o*，*m*，*p*-碘苯胺、*o*，*m*，*p*-硝基苯胺的分离因子和分离度等色谱参数。

**图8:**
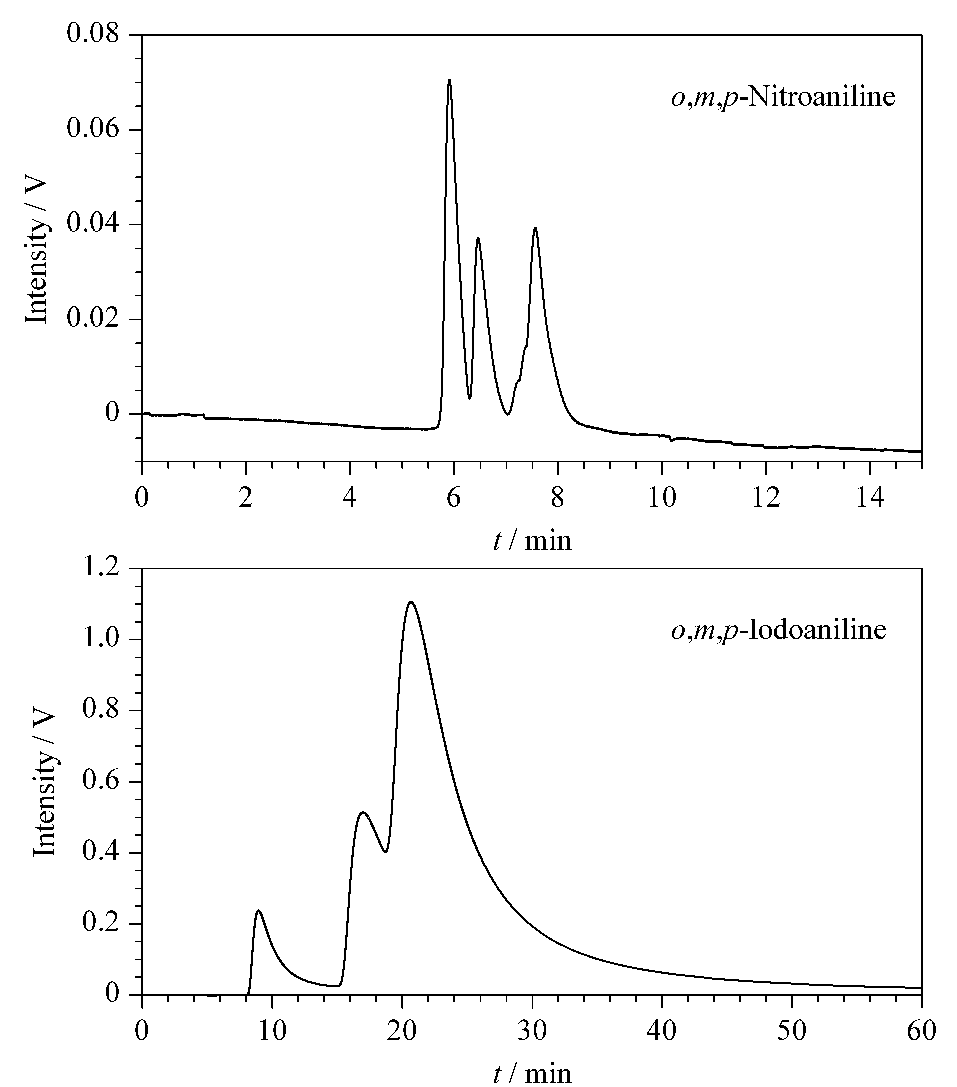
TpPa-NH_2_-Glu液相色谱柱对*o*，*m*，*p*-硝基苯胺和*o*，*m*，*p*-碘苯胺的分离色谱图

**表2:** TpPa-NH_2_-Glu液相色谱柱对于苯系位置异构体的拆分结果

Mobile phase	Racemate	*k*_1_	*k*_2_	*k*_3_	*α*_1，2_	*α*_2，3_	*R*_s1，2_	*R*_s2，3_
*n*-Hexane-isopropanol（9∶1，v/v）	*o*，*m*，*p*-nitroaniline	1.07	3.43	4.13	3.21	1.20	3.14	0.44
	*o*，*m*，*p*-iodoaniline	0.97	3.00	3.59	3.10	1.20	2.87	0.46

*k*_1_：the retention factor of the first peak；*k*_2_：the retention factor of the second peak；*k*_3_：the retention factor of the third peak；*α*_1，2_：the separation factor of the first and second peaks；*α*_2，3_：the separation factor of the second and third peaks；*R*_s1，2_：resolution of the first and second peaks；*R*_s2，3_：resolution of the second and third peaks.

### 2.5 温度对TpPa-NH_2_-Glu液相色谱柱的影响

为了研究不同温度对TpPa-NH_2_-Glu液相色谱柱出峰情况的影响，以华法林为例进行测试。探究了25、30、35、40和45 ℃ 5个温度对TpPa-NH_2_-Glu液相色谱柱出峰效果的影响。从图9可以看出，在其他条件下不变的情况下，保留时间随着温度的增加而增加，对液相色谱柱的出峰没有太大影响。

**图9:**
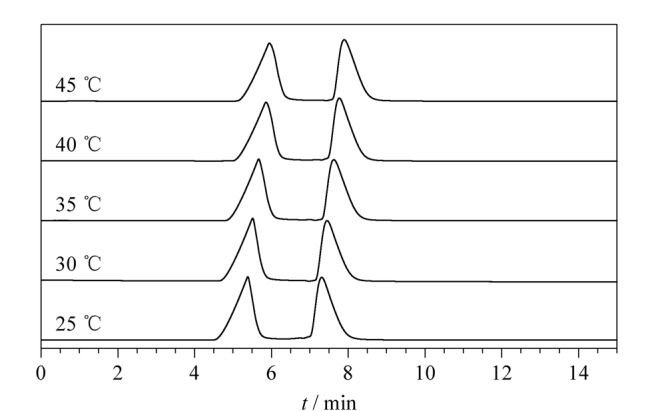
不同温度下华法林的色谱图

### 2.6 TpPa-NH_2_-Glu液相色谱柱的重复性

以分离效果较好的华法林为例，对制备的TpPa-NH_2_-Glu液相色谱柱的重复性进行考察。在流速为0.5 mL/min、柱温为25 ℃、流动相为甲醇-水（9∶1， v/v）的色谱条件下，该液相色谱柱经过5次重复测样后的拆分色谱图如图10所示。从图10可以看出，华法林的色谱拆分效果没有明显变化，测得的华法林的保留时间与峰面积的相对标准偏差分别为1.55%和1.46%。结果表明，用TpPa-NH_2_-Glu作为固定相填充的液相色谱柱具有良好的重复性。

**图10:**
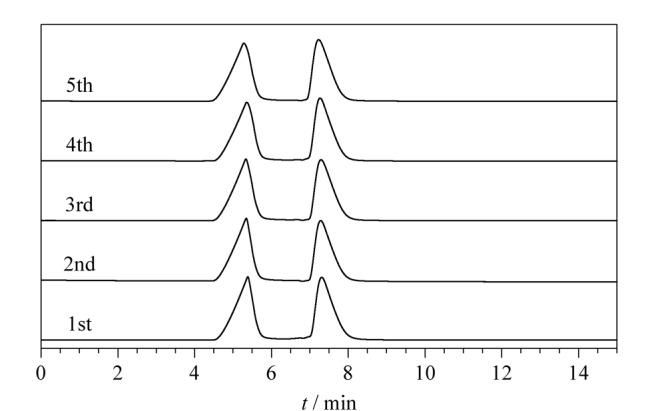
TpPa-NH_2_-Glu液相色谱柱的重复性分离谱图

## 3 结论

将手性TpPa-NH_2_-Glu用作高效液相色谱固定相，能对盐酸普萘洛尔、华法林、美托洛尔、2-溴丙酸乙酯、3-丁炔-2-醇手性化合物进行基线分离，对*o*，*m*，*p*-硝基苯胺、*o*，*m*，*p*-碘苯胺位置异构体具有良好的分离效果。

该研究表明，手性TpPa-NH_2_-Glu是一类很有发展潜力的手性分离材料，特别是对药物类的手性化合物分离分析方面有很大前景，对该类材料深入研究，将推进手性识别材料的发展。

## References

[1] Yuan L M. Chiral Recognition Material. 2nd ed. Beijing: Science Press, 2020

[2] Qian Z Y, Zhang M Y, Li S N, et al. Chinese Journal of Chromatography, 2018, 36(3): 261 30136504 10.3724/SP.J.1123.2017.11009

[3] Xie S M, Zhang Z J, Wang Z Y, et al. J Am Chem Soc, 2011, 133(31): 11892 21751804 10.1021/ja2044453

[4] Schmid A, Dordick J S, Kiener A, et al. J Chromatogr A, 2001, 409(2817): 258 10.1038/35051736 11196655

[5] Yuan H M, Lu Z Y, Li Y H, et al. Chinese Journal of Chromatography, 2022, 40(2): 109 35080157 10.3724/SP.J.1123.2021.04029 PMC9404014

[6] Diercks C S, Yaghi O M. Science, 2017, 355(6328): 1585 10.1126/science.aal1585 28254887

[7] Diercks C S, Kalmutzki M J, Yaghi O M. Molecules, 2017, 22(9): 1575 32961648 10.3390/molecules22091575 PMC6151596

[8] Yang C X, Yan X P. Chinese Journal of Chromatography, 2018, 36(11): 1075 30378369 10.3724/SP.J.1123.2018.06023

[9] Zhang W M, Liu K C, Ma W D, et al. Chinese Journal of Chromatography, 2022, 40(7): 600 35791598 10.3724/SP.J.1123.2021.12004 PMC9404040

[10] Han H X, Chu J Q, Wen Z W. Chin Chem Lett, 2022, 33(5): 2464

[11] Huang W, Jiang Y, Li X, et al. ACS Appl Mater Interfaces, 2013, 5(18): 8845 23927756 10.1021/am402649g

[12] Qian H L, Yang C X, Yan X P. Nat Commun, 2016, 7: 12104 27401541 10.1038/ncomms12104 PMC4945876

[13] Han X, Huang J J, Yuan C, et al. J Am Chem Soc, 2018, 140(3): 89 10.1021/jacs.7b12110 29302963

[14] Yuan C, Wu X W, Gao R, et al. J Am Chem Soc, 2019, 141(51): 20187 31789030 10.1021/jacs.9b10007

[15] Ma T T, Yang C, Qian H L, et al. J Chromatogr A, 2022, 1673: 463085 35500391 10.1016/j.chroma.2022.463085

[16] Chong J H, Sauer M, Patrick B O, et al. Org Lett, 2003, 5(21): 3823 14535719 10.1021/ol0352714

[17] Biswal B P, Chandra S, Kandambeth S, et al. J Am Chem Soc, 2013, 135(14): 5328 23521070 10.1021/ja4017842

